# A capital-based approach to understand health inequalities: empirical explorations

**DOI:** 10.1093/eurpub/ckac130.003

**Published:** 2022-10-25

**Authors:** Y Qi, JC Vrooman, J Almansa, P Ots, S Brouwer, SA Reijneveld

**Affiliations:** 1 Health Sciences, University Medical Center Groningen, Groningen, Netherlands; 2 Department of Sociology, ICS, Utrecht University, Utrecht, Netherlands; 3 The Netherlands Institute for Social Research, SCP, Den Haag, Netherlands

## Abstract

**Background:**

The persistence of health inequalities may not be merely driven by education and income, but also by other economic and non-economic factors. In this study we investigated how the association between single-dimensional health and socioeconomic status (SES) changes when including health-related person capital, economic capital, social capital, cultural capital and non-health related person capital.

**Methods:**

The present study proposes a capital-based approach to understand health inequalities. It presumes intertwined relationships between a wide notion of health (‘health-related person capital’) and embodied resources (‘non-health related person capital’) on the one hand, and non-person capital, i.e. economic, social, and cultural resources on the other. We used cross-sectional data on 152,592 participants from the Dutch Lifelines cohort study. Correlations between capital constructs were estimated using partial least squares structural equation modelling.

**Results:**

The correlation between health-related person capital and SES (r = 0.15) was higher than the correlations between single-dimensional health (physical and mental health) and SES (r = 0.12, r = 0.04, respectively). Non-person capital, combining economic, social and cultural capital, showed a correlation of 0.34 with health-related person capital. This was higher than the correlation between health-related person capital and economic capital alone (r = 0.19). Lastly, the correlation between health-related person capital and non-person capital increased when non-health (personality and attractiveness) and health related person capital were combined into person capital construct (from r = 0.34 to r = 0.49).

**Conclusions:**

This exploratory observational study shows the empirical interconnectedness of various types of resources. Our findings corroborate the idea of considering health as a multidimensional concept, and to extend conventional SES indicators to a broader measurement of economic and non-economic resources.

**Key messages:**

- A wide notion of capital may be key to a better understanding of the persistence of health inequalities.
- Policy action needs to take into account the unequal distribution of economic, social, cultural, and person capital.

